# Downregulation of endothelial nitric oxide synthase (eNOS) and endothelin-1 (ET-1) in a co-culture system with human stimulated X-linked CGD neutrophils

**DOI:** 10.1371/journal.pone.0230665

**Published:** 2020-04-06

**Authors:** Akari Nakamura-Utsunomiya, Miyuki Tsumura, Satoshi Okada, Hiroshi Kawaguchi, Masao Kobayashi

**Affiliations:** Department of Pediatrics, Hiroshima University Graduate School of Biomedical and Health Sciences, Hiroshima, Japan; National Institutes of Health, UNITED STATES

## Abstract

Phagocytes in patients with chronic granulomatous disease (CGD) do not generate reactive oxidative species (ROS), whereas nitric oxide (NO) production is increased in response to the calcium ionophore A23187 in CGD phagocytes compared with healthy phagocytes. Recently, patients with X-linked CGD (X-CGD) have been reported to show higher flow-mediated dilation, suggesting that endothelial cell function is affected by NO production from phagocytes. We studied *NOS3* and *EDN1* mRNA and protein expression in human umbilical vein endothelial cells (HUVECs) in a co-culture system with neutrophils from X-CGD patients. HUVECs were co-cultured for 30 minutes with human neutrophils from X-CGD or healthy participants in response to A23187 without cell-to-cell contact. The expression of *NOS3* and *EDN1* mRNA in HUVECs was quantified by real-time polymerase chain reaction. Moreover, we demonstrated the protein expression of eNOS, ET-1, and NFκB p65, including phosphorylation at Ser1177 of eNOS and Ser536 of NFκB p65. Neutrophils from X-CGD patients showed significantly higher NO and lower H_2_O_2_ production in response to A23187 than healthy neutrophils in vitro. Compared with healthy neutrophils, X-CGD neutrophils under A23187 stimulation exhibited significantly increased NO and decreased H_2_O_2_, and promoted downregulated *NOS3* and *EDN1* expression in HUVECs. The total expression and phosphorylation at Ser1177 of eNOS and ET-1 expression were significantly decreased in HUVECs co-cultures with stimulated X-CGD neutrophils. Also, phosphorylation at Ser536 of NFκB p65 were significantly decreased. In conclusions, eNOS and ET-1 significantly down-regulated in co-culture with stimulated X-CGD neutrophils through their excessive NO and the lack of ROS production. These findings suggest that ROS generated from neutrophils may mediate arterial tone affecting eNOS and ET-1 expression via their NO and ROS production.

## Introduction

Chronic granulomatous disease (CGD) is a rare, heterogenous, and inherited disorder that affects approximately 1 in 250,000 births [1]. It has been reported that X-linked CGD occurs in approximately 70% of patients with CGD and is due to the mutation of *CYBB* encoding gp91^phox^, which is located at Xp21.1 [2, 3]. NADPH oxidase activity is diminished in activated leukocytes obtained from these patients, leading to a reductions in reactive oxygen species (ROS) such as H_2_O_2_ and resulting in severe and recurrent bacterial and fungal infections. Among the mutational defects of the NADPH oxidase subunit complex, functional deficiency of gp91^phox^ is the most common, resulting in X-CGD [4]. It has already been reported that phagocytes in CGD patients do not generate ROS such as superoxide ions (O_2_^-^) and H_2_O_2_ under inflammatory stimulation (e.g., with lipopolysaccharide), whereas nitric oxide (NO) production by CGD phagocytes has been reported to be increased in response to a calcium ionophore, A23187, compared with that of phagocytes from healthy people [5–10].

In 2009, Violi et al. reported that lower oxidative stress and enhanced arterial dilatation as assessed by flow-mediated dilatation (FMD) testing were detected in X-CGD patients, reflecting increased bioavailability or higher levels of NO [11–13]. Their findings suggested that oxidative stress derived from neutrophils may have a pivotal role in modulating endothelial function [14,15]. However, the precise interactions between the NO and ROS produced by neutrophils in particular, and their effects on endothelial function, remain to be elucidated.

The endothelium is a regulator of vascular tone by releasing relaxing and contracting factors [16]. Among various endothelial-derived relaxing factors, the main species identified is NO, which is released in response to a variety of stimuli [17]. NO is a strong vasodilator and functions as a potent signaling molecule in many internal cells, including vascular endothelial cells [17, 18]. Among the three distinct isoforms of NO synthase (NOS), the relatively small amounts of NO produced by endothelial NOS (eNOS) are important for cardiovascular homeostasis, whereas the high NO levels associated with activated inducible NOS (iNOS) are related to infection and inflammation in vivo [19]. An excessive dose of NO is likely to induce endothelial damage. In addition, because NO is produced by NOS in several cell types, it can rapidly undergo a series of reactions with molecules such as oxygen and superoxide anions that inactivate NO [20]. Among these reactions, NO reacts more rapidly with O_2_^-^ to form peroxinitrite (ONO_2_^-^, which itself is strongly oxidizing) than with O_2_^-^ to form H_2_O_2_ [20]. It has also been reported that shear stress, which is important for inducing eNOS expression, stimulates increased eNOS (*NOS3*) transcription via activation of nuclear factor kappa B (NFκB) and binding of p50/p65 heterodimers to a shear responsive element in the human *NOS3* promoter [21].

In addition to NO, endothelin-1 (ET-1) has been considered as an essential molecules in the process of endothelial toning as well as eNOS [22–24]. There are several reports indicating that NO has a role in the inhibitory regulation of ET-1 (*EDN1*) production at the transcription level in endothelial cells [25–27]. In vascular systems, *NOS3* and *EDN1* cooperate as a toning-modulator molecules with opposing roles.

In this study, we hypothesized that the gp91^phox^ subunit of NADPH oxidase derived from neutrophils could have a significant effect on endothelial function. To investigate this potential effect, we focused on the effect of NO and H_2_O_2_ from neutrophils obtained from patients with X-CGD on the expression of *NOS3* and *EDN1* mRNA in human umbilical vein endothelial cells (HUVECs). In addition, we demonstrated that the protein expression of eNOS, ET-1, phosphorylated eNOS at Ser1177 position, total NFκB at p65, and phosphorylated p65 at Ser536.

## Materials and methods

### Study population

The study protocol was approved for use by the Human Studies Subcommittee of the Hiroshima University Graduate School of Biomedical and Health Sciences. For participation in this study, the doctor in charge informed each participants and their guardians by written format about the consents for use of study samples for research and the use of their medical records. We gained the consent provided by participants and/or their parents/guardians was written. Blood samples were collected after obtaining informed consent from all patients and participants. Table 1 shows the clinical characteristics of 20 X-CGD patients and 20 age-matched healthy male participants (controls) who were enrolled from April 2009 to September 2015. The diagnosis of X-CGD was made based on flow cytometry analysis of intracellular 7D5 proteins, a previously reported method using a monoclonal antibody raised against the human flavocytochrome b558 [28, 29].

**Table 1:** Clinical characteristics of X-CGD patients and healthy participants (controls).

Variable	CGD patients (n = 20)	Controls (n = 20)	P value
Age, years	13±10	10±10	NS
Body mass index, kg/m^2^	16.8±3.5	18.1±3.6	NS
Systolic blood pressure, mm Hg	106±10	102±12	NS
Diastolic blood pressure, mm Hg	58±10	50±8	NS
White blood cell count, /μL	7830±3450	7280±3200	NS
Neutrophil count, /μL	4690±3260	3580±1740	NS
C-reactive protein, mg/dl	0.63±0.64	0.07±0.10	<0.01
Total cholesterol, mg/dl	190±111	161±43	NS
Triglyceride, mg/dl	140±88	108±40	NS
Medication			
Antibiotics, n (%)	14 (70)	0 (0)	<0.01
Immune-suppression drug, n (%)	3 (15)	0 (0)	<0.01

All CGD patients were examined for 7D5 antibodies using by FACS analysis. All the study subjects were men. Statistical analysis was conducted using an unpaired two group t-test. *P* < 0.05 was considered significant.

### Preparation of neutrophils

Human neutrophils were prepared as previously described [30]. In brief, 5 mL blood samples were diluted with an equal volume of 6% dextran solution for 30 minutes, from which approximately 4 mL of low-density solution was carefully harvested, layered onto 3 mL Lymphoprep, and subjected to centrifugation at 800×g for 10 mins. The cells were resuspended with 5 mL lysis buffer for 5 minutes. After washing with phosphate-buffered saline (PBS) and subjecting to centrifugation at 800×g for 10 mins, the supernatant was discarded. The lysis buffer segmentation procedure was repeated three times in total. The neutrophil preparation was subjected to a final centrifugation and suspended in 1 mL PBS at room temperature.

### Flow cytometric analysis of DAF2/DA-positive neutrophils

We conducted fluorescence-assisted cell sorter (FACS) analysis as follows with reference to a previously described method [9]. A total of 1 ×10^5^ neutrophil cells diluted in 1 mL PBS were stimulated with 2 μM A23187 (Sigma Chemical Co., St. Louis, MO, USA) for 20 mins in vitro, then co-incubated with DAF/2DA reagent (Sigma Chemical Co.), a fluorescent indicator of intracellular NO production, at 10 μM for 20 mins [31]. Stained cells were analyzed at the start and after 60 mins period of analysis using a FACS Calibur system (Becton–Dickinson Immunocytometry Systems, San Jose, CA, USA). More than 1 ×10^4^ cells were counted and analyzed by FACS in each experiment.

### Nitrite and nitrate measurement

The concentration of total nitrite and nitrate (the final products of NO) in the supernatant of the co-culture of HUVECs with neutrophils diluted with Hank’s balanced salt solution (HBSS; Nissui, Tokyo, Japan) were measured using a colorimetric assay kit (Cayman Chemical Company, Ann Arbor, MI, USA) based on the Griess method, which converts nitrite into an azo chromophore for accurate determination of the NO_2_^-^ concentration. Each sample was recorded in duplicate wells.

### H_2_O_2_ measurement

A colorimetric assay kit (Bio Vision, Milpitas, CA, USA) was used to determine the H_2_O_2_ concentration in the supernatant of co-cultures of neutrophils and HUVECs. Samples were filtered through a 10 kDa MW spin filter to remove all proteins then stored at −80°C. In the presence of horseradish peroxidase, the OxiRed probe reacts with H_2_O_2_ to generate colored products that can be measured by optical density (550 nm) in a microplate reader. Each sample was recorded in duplicate wells and the concentration of H_2_O_2_ was calculated by applying the samples to standard curves.

### Cell culture

HUVECs were purchased from Lonza (Basel, Switzerland). Confluent cells cultured for 5–7 days were used for the analysis. Purified cells (1–2 ×10^4^) were cultured in a 12-well transwell system with a polycarbonate insert membrane (pore size: 0.4 μm) (Corning Coaster Inc., Corning, NY, USA) in accessory medium (1 μL/mL human epidermal growth factor, 2 μL/mL hydrocortisone, 2% fetal bovine albumin, 1 μL/mL vascular endothelial growth factor, 4 μL/mL human fibroblast growth factor B, 1 μL/mL insulin-like growth factor 1, 1 μL/L ascorbic acid, 1 μL/mL heparin, and 1 μL/mL GA-1000) and used for real-time polymerase chain reaction (PCR). Incubation was performed at 37°C in a humidified atmosphere with 5% CO_2_/95% air. When HUVECs were used, the culture medium was changed to HBSS at 2 hours prior to each analysis.

### O_2_^-^ and NO generation assay

We performed the H_2_O_2_ and NO generation assays from two culture systems: a neutrophil-free culture system constructed from reagents, and a neutrophil-containing culture system. For the neutrophil-free cultures, we added the following reagent combinations into wells containing confluent HUVECs: 1) xanthine and xanthine oxidase (H_2_O_2_ generation system: X/XO); 2) NO donor S-nitrosoglutathione (NO generation system: NO); and 3) xanthine and xanthine oxidase with NO donor (mixed system of H_2_O_2_ and NO: X/XO+NO). In the H_2_O_2_-generating culture systems, 4 mM xanthine (Sigma Chemical Co.) and 12.5 U/mL xanthine oxidase (Sigma Chemical Co.) on an insert membrane were added to HUVECs cultured in 1 mL HBBS. NO generation was assayed with the addition of the NO donor S-nitroso-N-acetyl-DL-penicillamine (SNAP; Dojindo Laboratories, Kumamoto, Japan) at 3 μM in HUVEC cultures. Neutrophil- containing cultures were assayed with or without 10 μM A23187 added to 1 ×10^5^ neutrophils on an insert membrane added to HUVEC cultures. HUVECs and neutrophils did not have direct contact in the co-culture wells. The co-culture duration used for real-time PCR samples was 30 minutes.

### Reverse Transcriptase (RT)-PCR and quantitative real-time PCR

Total cellular RNA was extracted from cultured HUVECs using the Isogen extraction method, and cDNA was synthesized from 0.5 μg total RNA using a first-strand synthesis system for RT-PCR (ReverTra Ace-α; TOYOBO, Osaka, Japan). The *NOS3 and EDN1* primer sequences used for real-time PCR were as follows: *NOS3* forward AGATCTCCGCCTCGCTCAT and reverse CATACAGGATTGTCGCCTTCAC, and *EDN1* forward ACTTCTGCCACCTGGACATC and reverse GGCAAAAATTCCAGCACTTC.

After cDNA was synthesized and amplified, the products were analyzed and the density was scanned. The products of real-time PCR were normalized with reference to the values obtained for the endogenous *YWHAZ* cDNA. Human *YWHAZ* primers (human housekeeping gene primer set) were purchased from Takara Bio (Tokyo, Japan).

### Western blot analysis of eNOS, ET1, NfκB p65, phosphorylated eNOS (Ser1177 position) and phosphorylated NfκB p65 (Ser536 position)

HUVECs (1×10^6^/well) were seeded in 6-well plate and exposed to reagents and neutrophils conditions as follows. In the reagents system, 4 mM xanthine, 12.5mU/ml xanthine oxidase (X/XO), and 3 mM NO-donor (NO) were added to the appropriate wells for 0.5 hrs. In- neutrophils-containing wells, 1×10^6^ neutrophils were incubated with or without 10 μM A23187 for 0.5 hrs. The cells were harvested with RIPA buffer and used for samples. Proteins (120 μg) were resolved by nitrocellulose gel electrophoresis and electroblotted onto PVDF membranes (Millipore, Billerica, MA, USA). For immunodetection, membranes were blocked in 5% BSA in TBS with 0.1% Tween-20 (TBST) and incubated in TBST with eNOS and phosphorylated-eNOS(Ser1177) antibodies (Cell life signaling, Danvers, MA, USA) (1:100 dilution), anti-EDN1 antibody (Sigma-Aldrich, St. Louis, MO, USA) (1:500), anti- NFκB (p65) antibody, and anti-phosphorylated NFκB (p65) at Ser536 position (St. John’s Laboratory, London, UK) (1:500), and anti-GAPDH antibody (Sigma-Aldrich, St. Louis, MO, USA) (1:1000) with chemiluminescent detection. Blots were incubated with peroxidase-labeled anti-rabbit or anti-mouse IgG secondary antibody (1:20,000; cat# PI-1000 (Rabbit), PI-2000 (mouse), Vector Labs, Burlingame, CA, USA) at room temperature, followed by enhanced chemiluminescence detection (Immobilon Western HRP substrate Luminol Reagent, Millipore, Billerica, MA, USA). The chemiluminescent reaction was analyzed by Versa Doc MP4000 (Bio Rad Laboratories, Inc, Hercules, CA, USA). We quantified the each band using Image J software (National Institutes of Health, MD).

### Statistical analysis

All values are reported as means ± standard error from five independent experiments. Each experiment included duplicate measurements for each condition. The statistical significance of the data was determined with analysis of variance or unpaired two-group *t*-tests using SPSS 19.0 (IBM Corp., Armonk NY, USA). A *P*-value < 0.05 was considered significant for all tests.

## Results

### *NOS3* mRNA and protein levels of total and phosphorylated eNOS at Ser1177 are significantly decreased in HUVECs co-cultured with X-CGD neutrophils

**Fig 1:**
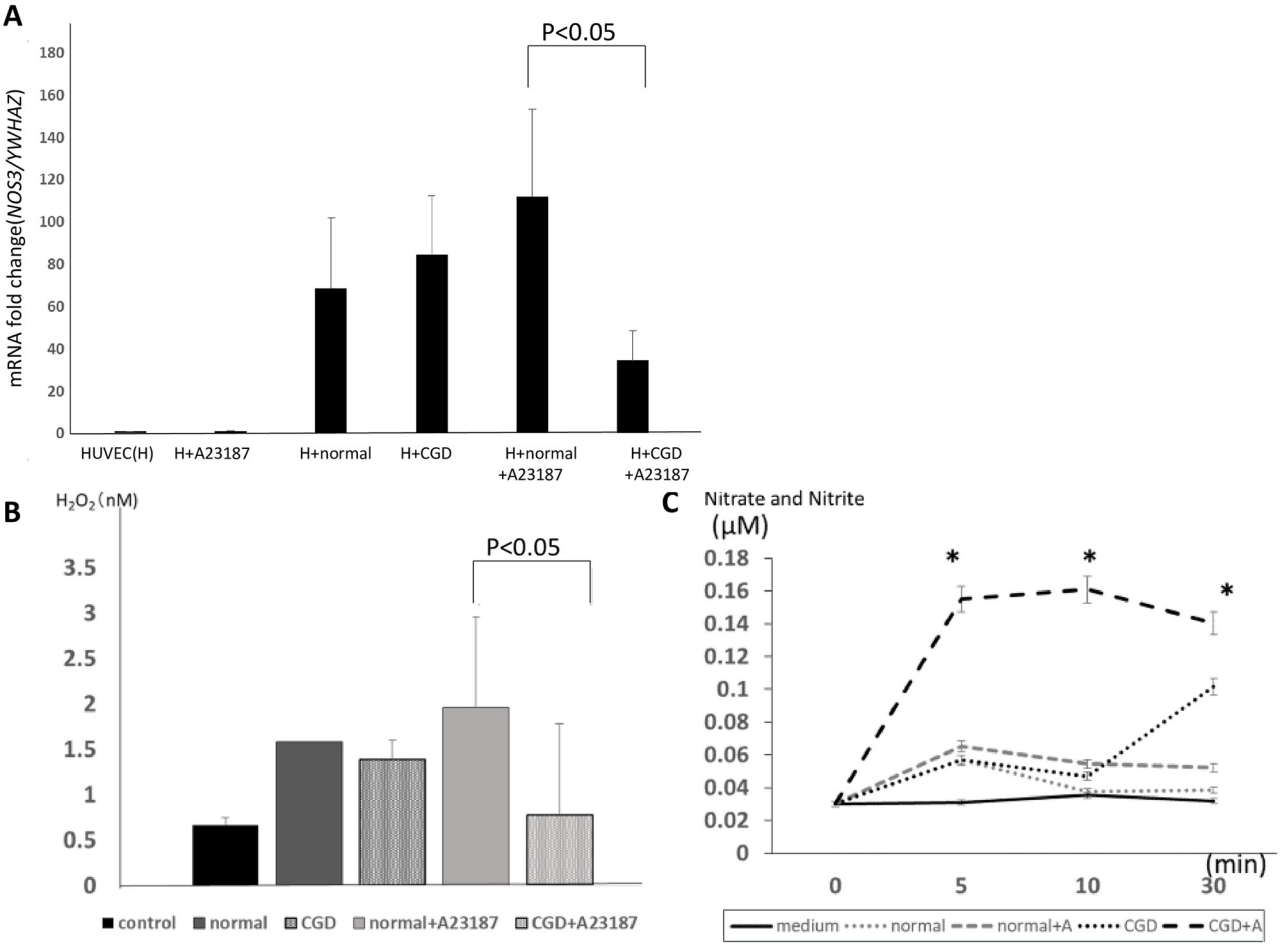
mRNA levels of NOS3 in HUVECs in neutrophil-containing culture. (A) Expression of *NOS3/YWHAZ* mRNA by RT-PCR using HUVECs alone as a relative control (ratio = 1). (B) H_2_O_2_ concentration with and without A23187 stimulation in the supernatants of HUVECs co-cultured with X-CGD neutrophils for 30 minutes compared with healthy control neutrophils. (C)Total nitrate and nitrite concentration in the supernatant of co-cultures of HUVECs incubated with healthy control and X-CGD neutrophils for 30 minutes with and without A23187 stimulation. *P<0.05 compared with the medium and normal neutrophils +A23187 group.

**Fig 2:**
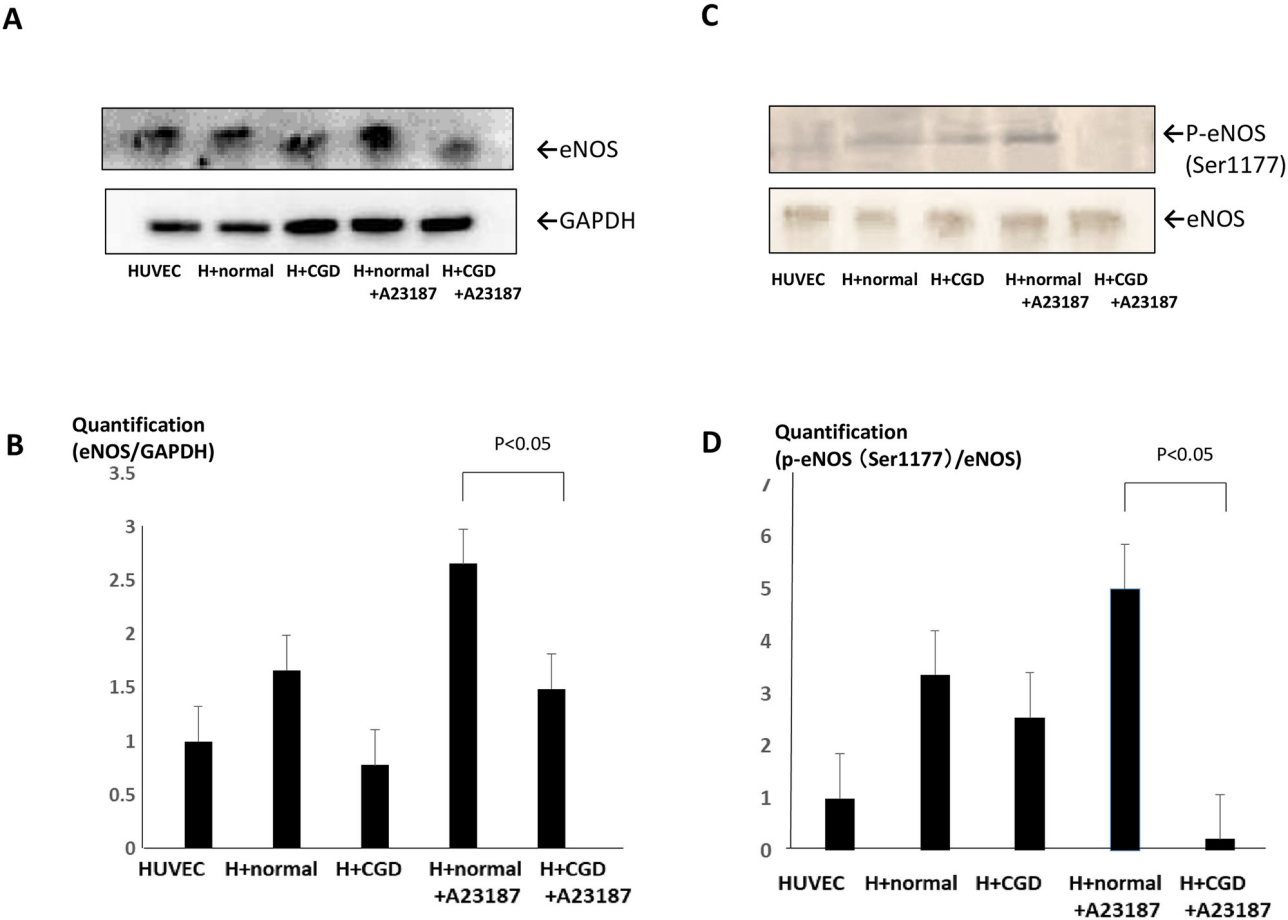
eNOS protein levels in HUVECs in neutrophil-containing culture. (A): Total protein expression of eNOS in HUVECs was examined by western blot analysis. Representative blots are shown. (B) Quantification of eNOS compared with GAPDH by Image J Software. *P<0.05 compared with the HUVECs with normal neutrophils +A23187 group. (C) The phosphorylated of eNOS at Ser1177 position in HUVECs was examined by western blot analysis. (D) Quantification of phosphorylated eNOS at Ser1177 compared with total eNOS protein by Image J Software. *P <0.05 compared with the HUVECs with normal neutrophils +A23187 group.

*NOS3* mRNA and the protein expression of phosphorylated eNOS at Ser1177 position in HUVECs co-cultured with X-CGD neutrophils are shown in Figs 1A and 2. To examine the effect of stimulated neutrophils on *NOS3* mRNA expression, HUVECs were cultured with neutrophils in a transwell-style permeable support system to avoid cell-to-cell contact. As shown in Fig 1A, when HUVECs were co-cultured with neutrophils under A23187 stimulation, the expression of *NOS3* mRNA was significantly decreased by nearly one-third in the presence of X-CGD neutrophils compared with healthy neutrophils. The H_2_O_2_ concentration in the supernatants of HUVECs cultured with A23187-stimulated X-CGD neutrophils was significantly lower compared with healthy neutrophils at 30 mins (Fig 1B). Moreover, the nitrate and nitrite concentration in the supernatants of HUVECs incubated with A23187-stimulated X-CGD neutrophils were significantly higher than that in the healthy control neutrophils, especially over 10 minutes of incubation (Fig 1C). These findings suggested that an increased level of NO persisting for more than 10 mins of co-incubation with A23187-stimulated X-CGD neutrophils induced the downregulation of *NOS3* mRNA expression.

To validate the mRNA changes, we further demonstrated the protein levels of eNOS and phosphorylated eNOS at Ser1177 position which indicates the activation of eNOS protein (Fig 2). As shown in Fig 2A, significant differences in total eNOS protein expression was detected between cells co-cultured with X-CGD and normal neutrophils. Moreover, phosphorylated eNOS at Ser1177 was significantly decreased in the samples co-cultured with X-CGD neutrophils (Fig 2B).

This finding suggested that the mechanism in *NOS3* mRNA is likely to associate with the activation of eNOS activity such as phosphorylation of eNOS at Ser1177 position, which is consistent with the results of previous reports [32].

### *EDN1* mRNA and protein levels of total ET-1 are decreased in HUVECs co-cultured with X-CGD neutrophils

**Fig 3:**
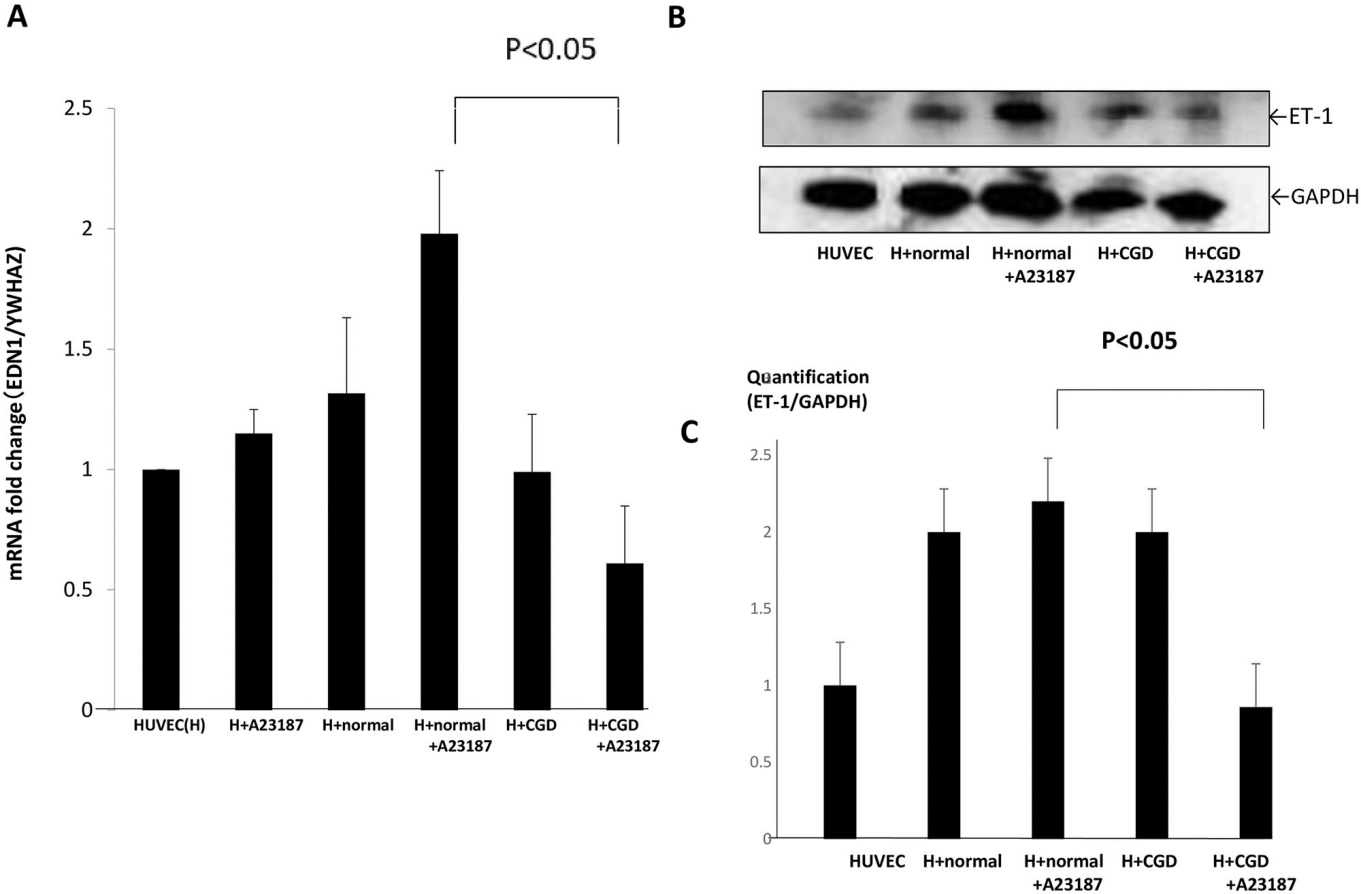
mRNA levels of *EDN1 and* ET-1 protein levels in HUVECs in neutrophil-containing culture. (A) Expression of *EDN1/YWHAZ* mRNA in HUVECs was examined by RT-PCR and real-time PCR using HUVECs alone as a relative control (ratio = 1). (B) ET1 protein expression and GAPDH in HUVECs in neutrophil-containing transwell system cultures. Representative blots are shown. Arrow at approximately 25kDa indicates ET-1. (C) Quantification of ET-1 bands compared with GAPDH expression. *P<0.05 compared with the HUVECs with normal neutrophils +A23187 group.

We demonstrated the *EDN1* mRNA changes and ET-1 protein levels were similar to *NOS3* in the neutrophils culture system. As shown in Fig 3A, decreased *EDN1* mRNA levels were significantly detected in HUVECs co-cultured with stimulated X-CGD neutrophils. To validate the mRNA changes, we further analyzed the protein levels of ET-1 and performed densitometry of each band (Fig 3B). HUVECs co-cultured with stimulated X-CGD neutrophils showed significantly decreased ET-1 expression compared with HUVECs with normal neutrophils as shown in Fig 3C.

### Phosphorylation at Ser536 of NFkB p65 protein is significantly decreased in HUVECs with stimulated X-CGD neutrophils

**Fig 4:**
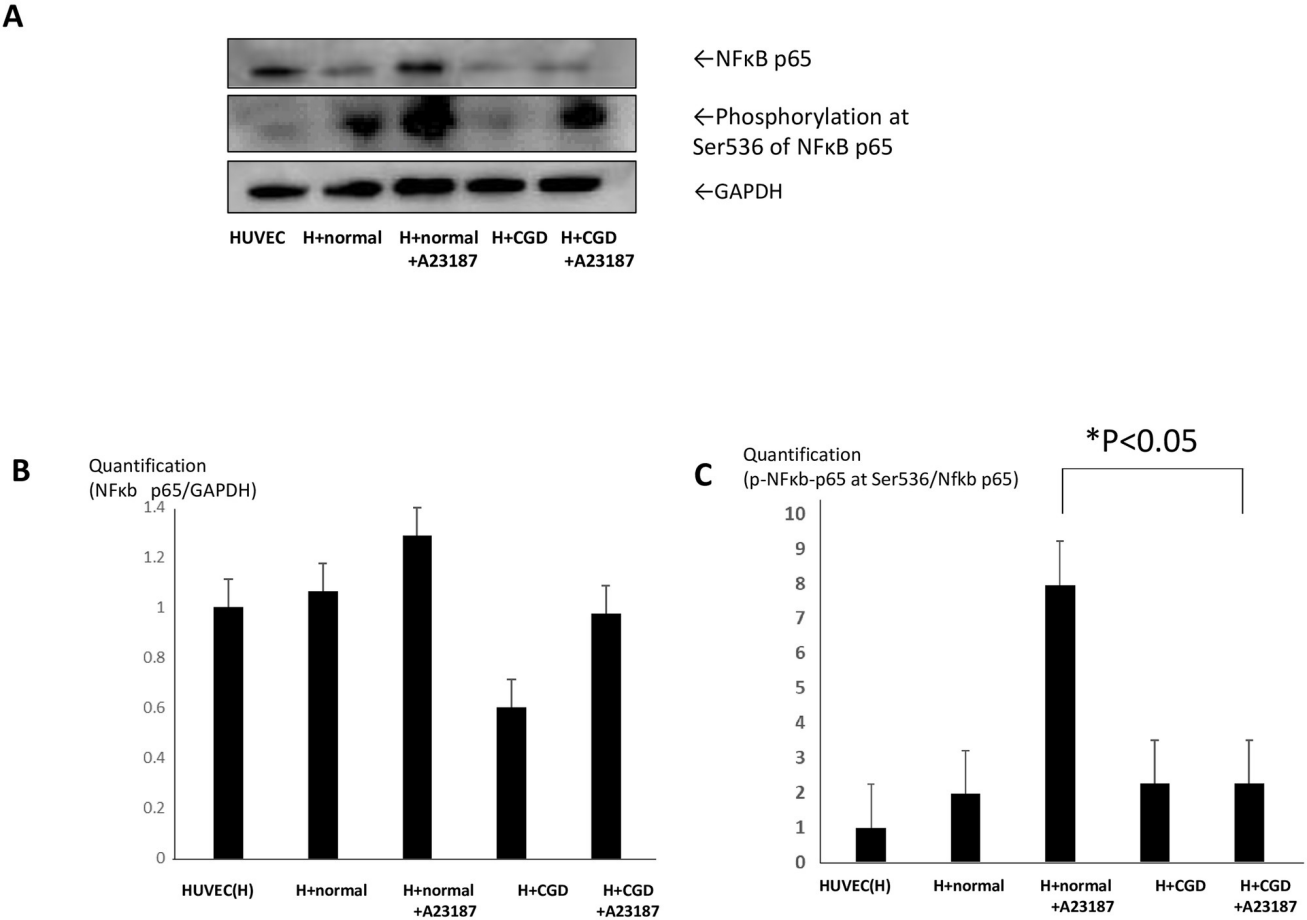
Total NFkB p65 protein and NFkB p65 phosphorylated at Ser536. (A) Total protein expression of NFκb p65 and phosphorylated at Ser536 in HUVECs in the neutrophil-containing transwell system compared with GAPDH expression. (B) Quantification of total NFκb p65. (C) Quantification of phosphorylation at Ser536 position compared with total NFκb p65.

We further demonstrated the phosphorylated NFκB p65, which indicates activation of the NFκB pathway, to evaluate the mechanism of the changes in eNOS and ET1 expression. Total NFκB p65 tended to be increased only in HUVECs with stimulated normal neutrophils (Fig 4B). In addition, phosphorylation at Ser536 of NFκB p65 was significantly decreased in HUVECs with stimulated X-CGD compared with stimulated normal in the neutrophil-culture system (Fig 4C). These findings suggested that the mechanism of *NOS3* and *EDN1* expression is likely to be related to the NFκB pathway, which is consistent with previous reports [21, 33].

### Increased NO from X-CGD neutrophils stimulated by A23187 is associated with the decreased of H_2_O_2_ production in vitro

**Fig 5:**
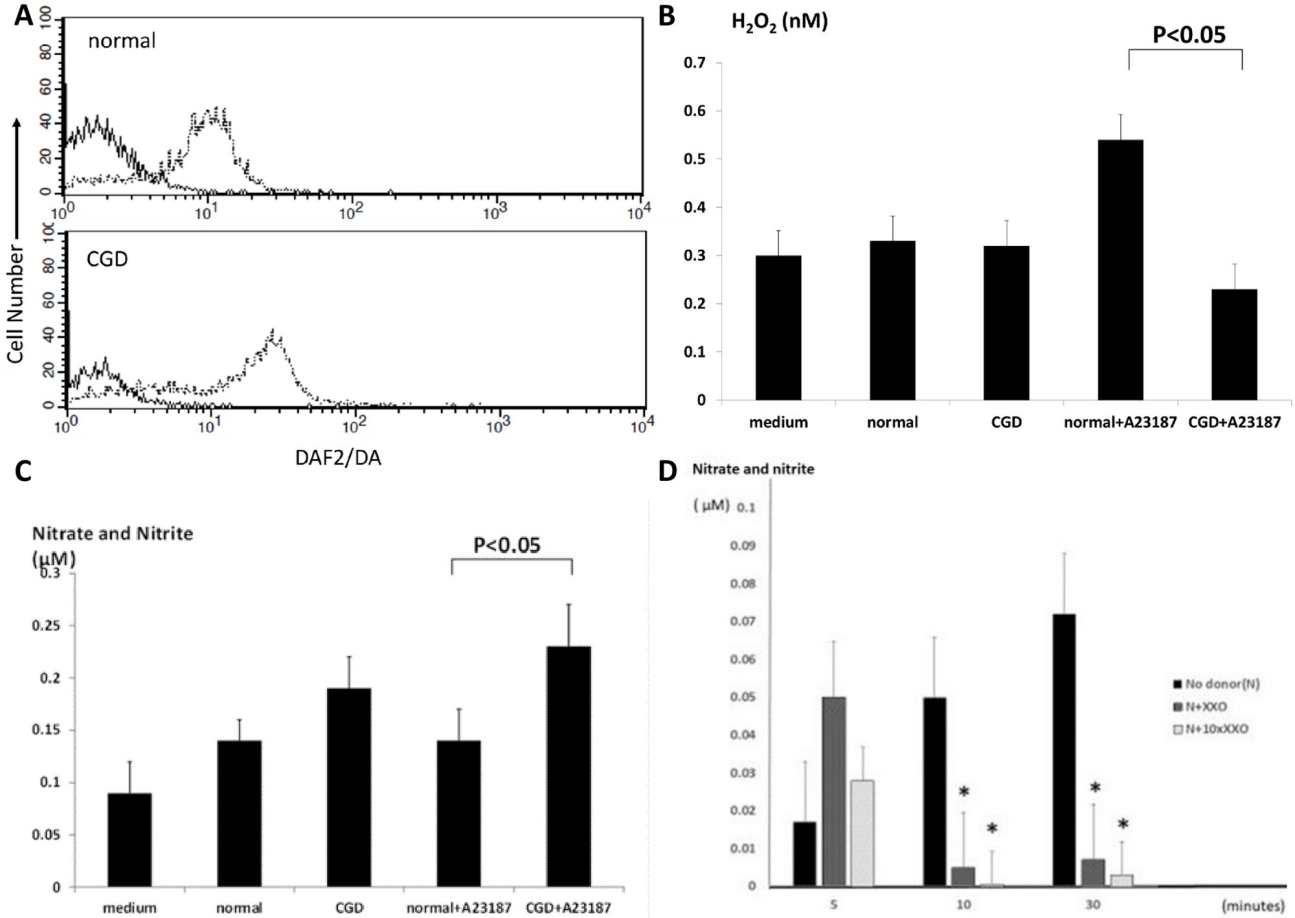
Increased NO from X-CGD neutrophils stimulated by A23187 is associated with the decreased of H_2_O_2_ production. (A) Representative FACS analysis data showing the quantification of NO-positive neutrophils in response to A23187 stimulation as a histogram of DAF2-DA-positive cells in the FL1 channel, which are detected at around 525 nm. The bold line shows the data before administration of A23187 and the thin line shows data taken 60 minutes after the addition of A23187. Upper panel, healthy participant; bottom panel, X-CGD patient. (B) H_2_O_2_ production in the supernatants of cultured neutrophils after 30 mins of incubation in vitro. (C) Total nitrate and nitrite production in the supernatants of cultured neutrophils after 30 mins of incubation in vitro. Data are expressed as mean ± SE. (D) Nitrate and nitrite concentration in culture media containing NO donors, xhantine and xhantine oxidase (X/XO) added at 5, 15, and 30 minutes. The concentration of NO metabolites at 5 mins tended to be higher when the NO donor was added with X/XO but was not significantly different between each sample. *P<0.05 compared with the NO group at each time period.

To confirm the NO and H_2_O_2_ production derived from neutrophils, we used FACS to analyze intracellular NO metabolism in neutrophils stimulated with A23187. Fig 5A shows a representative flow cytometric assay using DAF2-DA to detect the intracellular NO in neutrophils of patients with X-CGD and healthy controls before and after A23187 administration. After 20 mins of stimulation with A23187, the fluorescence of X-CGD neutrophils demonstrated a higher intensity than that of the normal neutrophils at 60 mins stimulation. (Fig 5A).

To confirm the differences in extracellular H_2_O_2_ and NO concentrations between healthy and X-CGD neutrophils, we measured the H_2_O_2_ and NO concentration produced from neutrophils in vitro. Fig 5B and 5C shows the concentrations of extracellular H_2_O_2_ and the final products of NO (total nitrate and nitrite) in the supernatants of neutrophils stimulated with A23187. The healthy neutrophils under stimulation generated significant levels of H_2_O_2_, while the X-CGD neutrophils failed to produce a similar level of H_2_O_2_, and the X-CGD neutrophils failed to produce a similar level of H_2_O_2_ (Fig 5B). There was no difference between the X-CGD and healthy control neutrophils in the production of total nitrite and nitrate in the absence of A23187. Under stimulation with A23187, however, X-CGD neutrophils produced significantly higher levels of extracellular nitrite and nitrate than the control neutrophils (Fig 5C). These findings were consistent with a previous report [10].

To verify the phenomenon that X-CGD neutrophils produced excessively high levels of NO compared with control neutrophils due to a lack of ROS, we measured the NO concentration produced artificially by a NO donor, with or without ROS generated by xanthine and xanthine oxidase. Fig 5D shows the change in total NO metabolites during the 30 minutes following administration of a NO donor: the nitrate and nitrite concentrations gradually increased, reaching a plateau at 10 mins. When ROS (xanthine and xanthine oxidase) were added, the nitrate and nitrite concentrations in the N+XXO and N+10XXO groups were significantly suppressed to almost 10% of the level of the maximum concentration of NO at 10 and 30 mins. (Fig 5D). These findings support the condition of cells lacking the capacity to generate substantial ROS that leads to prolonged and excessive levels of NO that escape inactivation by ROS. These results are also consistent with the suggestion that the lack of ROS generation in X-CGD neutrophils leads to the presence of prolonged, excessive NO in response to A23187 stimulation.

### In vitro HUVEC studies

#### Upregulation of *NOS3* and EDN1 mRNA by H_2_O_2_ administration and NO administration suppresses NOS3 and *EDN1* mRNA in neutrophil-free cultures

**Fig 6:**
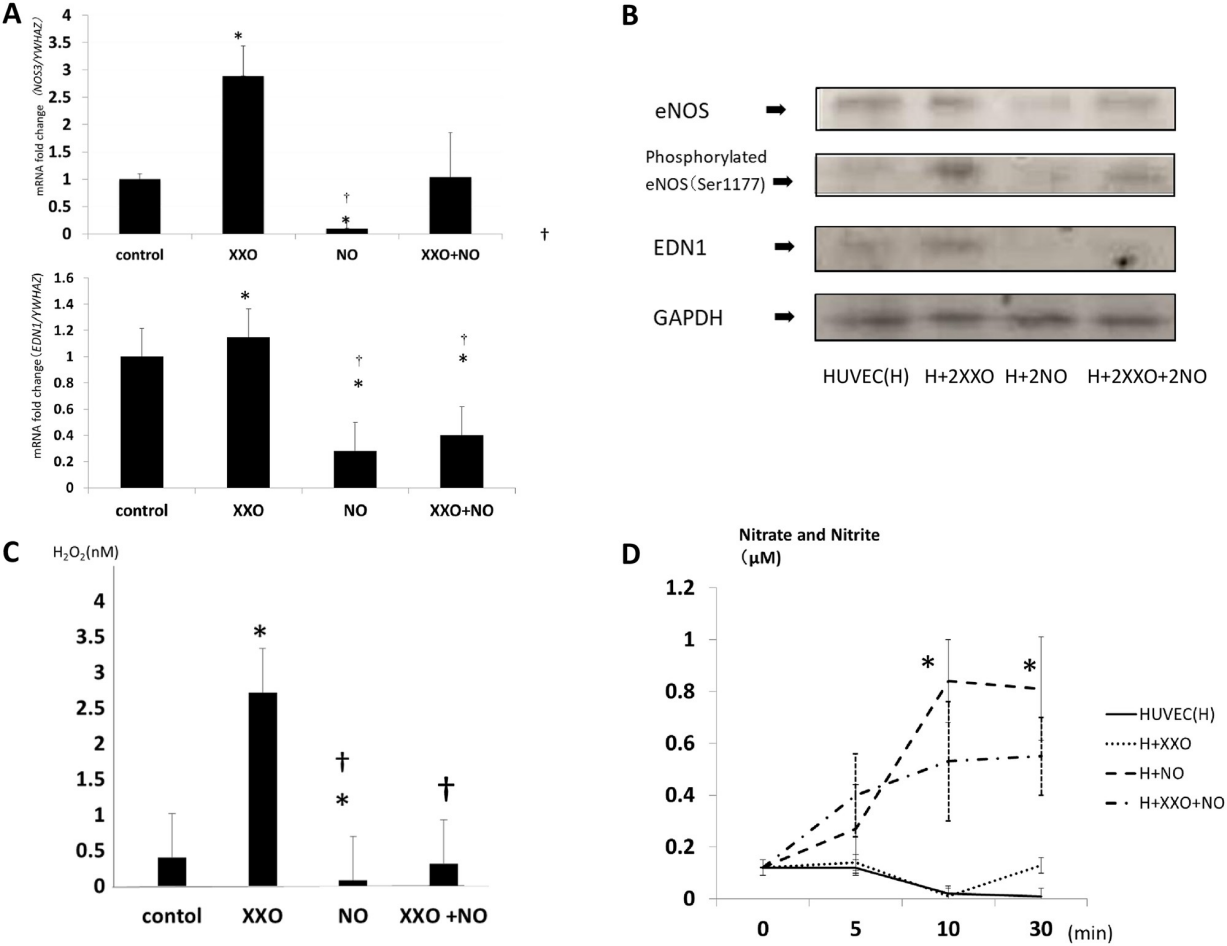
*NOS3* and *EDN1* expression in HUVECs in neutrophil-free culture. (A) *NOS3* and *EDN1* mRNA expression in HUVECs cultured in a neutrophil-free system. HUVECs alone were used as a relative control (ratio = 1). *P < 0.05 compared with control; +*P* < 0.05 compared with the 3-minute data in the same dose group. (B) Protein expression of eNOS, phosphorylated eNOS, ET-1, and GAPDH in a neutrophil-free culture with the addition of double-dose of X/XO and NO reagent. (C) The H_2_O_2_ concentration in the supernatant of HUVECs in neutrophil-free culture at 30 minutes was significantly higher in the X-XO group compared with the control, and significantly lower in the NO group compared with that in the control and X/XO groups. *P<0.05 compared with control; †*P*<0.05 compared with X/XO. (D) The total nitrate and nitrite concentrations in the supernatants were significantly increased in the presence of NO donor compared with that in HUVEC-only cultures at 10 mins and 30 mins. *P<0.05 compared with control.

To confirm the effect of ROS and NO on *NOS3* expression in endothelial cells, we constructed a neutrophil-free culture system using ROS-and/or NO-producing reagents. *NOS3* and *EDN1* mRNA expression in HUVECs was significantly upregulated under ROS generation in the X/XO system, consistent with a previous report [18,24]. In contrast, *NOS3* and *EDN1* mRNA expression was significantly downregulated in the NO generation system (NO), compared with that in medium alone (Fig 6A). When HUVECs were exposed to both the X/XO and NO generation systems, *NOS3* and *EDN1* expression was almost similar to the level in HUVECs alone (Fig 6A).

To validate this phenomenon, we demonstrated the eNOS and ET-1 protein expression in HUVECs co-cultured with a double-dose of each reagent (Fig 6B).

The H_2_O_2_ concentration at 30 minutes in this culture system is shown in Fig 4C. The X/XO system produced significantly more H_2_O_2_ than the control, NO and X/XO+ NO systems. In contrast, the NO donor-containing culture exhibited a lower H_2_O_2_ concentration measured from culture supernatants of the cell-free system, which increased with the addition of NO donor, and plateaued at 10 mins (Fig 6D). When the ROS-generating system was added, the nitrate and nitrite concentrations were significantly suppressed to almost half the level of the maximum concentration of NO. These findings indicated that extracellular ROS induced the upregulation of *NOS3* and *EDN1* mRNA and their protein expression, while extracellular NO administration resulted in a decrease in H_2_O_2_ in the medium, which led to the downregulation of *NOS3* and *EDN1* mRNA and their protein expression.

#### NO- and H_2_O_2_-dependent regulation of *NOS3* and *EDN1* expression in HUVECs in the cell-free culture system

**Fig 7:**
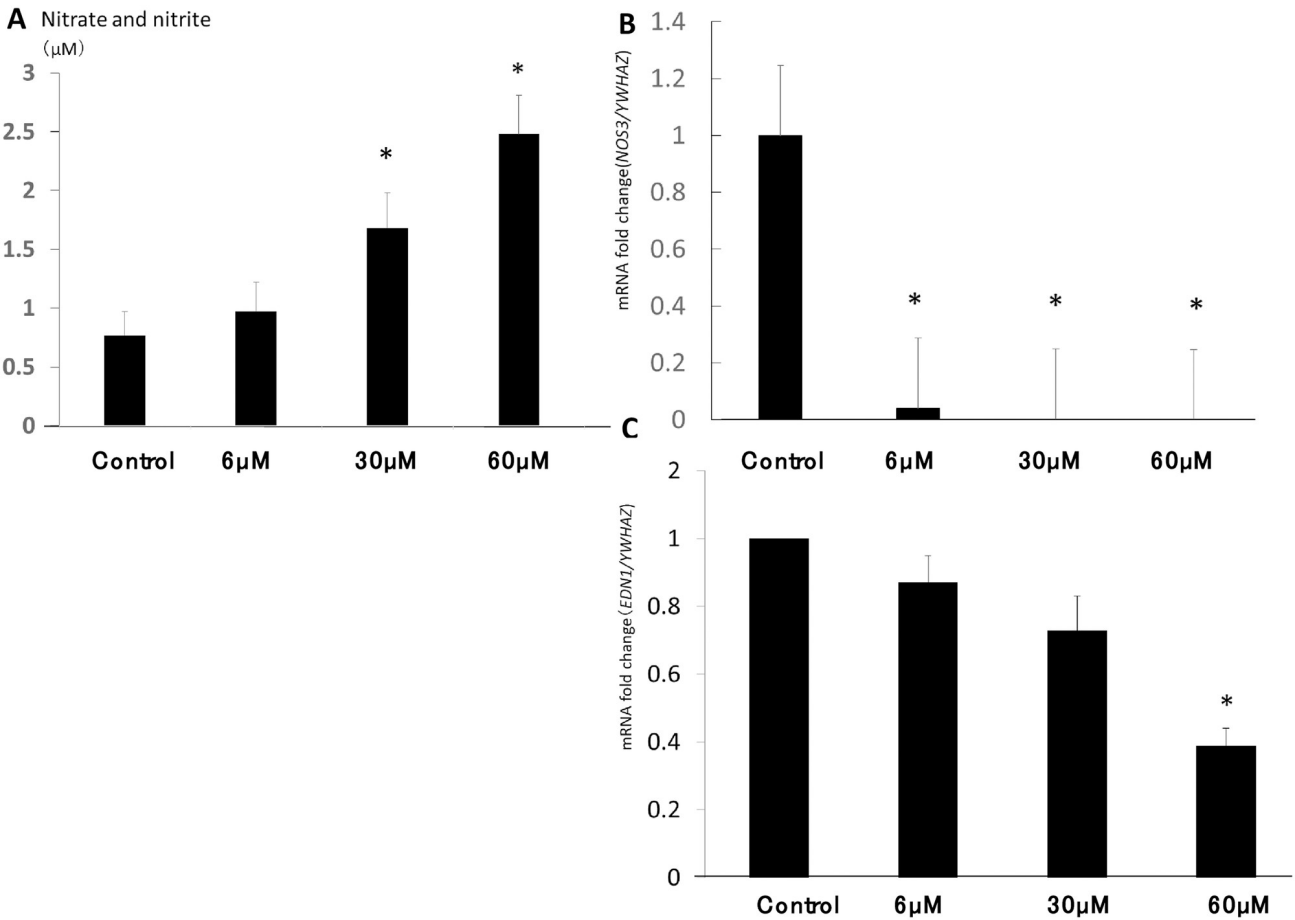
Effect of the administration of NO on endothelial *NOS3* and *EDN1* mRNA expression in HUVECs. NO donor S-nitrosoglutathione (0, 6, 30, 60 μM/well) was added to HUVECs (2 × 10^4^/well). **(A)** Nitrate and nitrite concentrations in the supernatant. **(B)** Quantitative real-time PCR analysis of *NOS3* expression in HUVECs. **(C)** *EDN1* mRNA expression. *P < 0.05 compared with control.

We examined the effects of NO concentration on *NOS3* and *EDN1* expression in HUVECs. As shown in Fig 7A, the nitrate and nitrite concentration in the supernatant of HUVEC culture increased with the addition of NO donor in a dose-dependent manner. The level of suppression of both *NOS3* and *EDN1* expression in HUVECs was dependent on the nitrate and nitrite concentrations in the supernatant, suggesting that NO inhibits the expression of *NOS3* and *EDN1* (Fig 7B).

**Fig 8:**
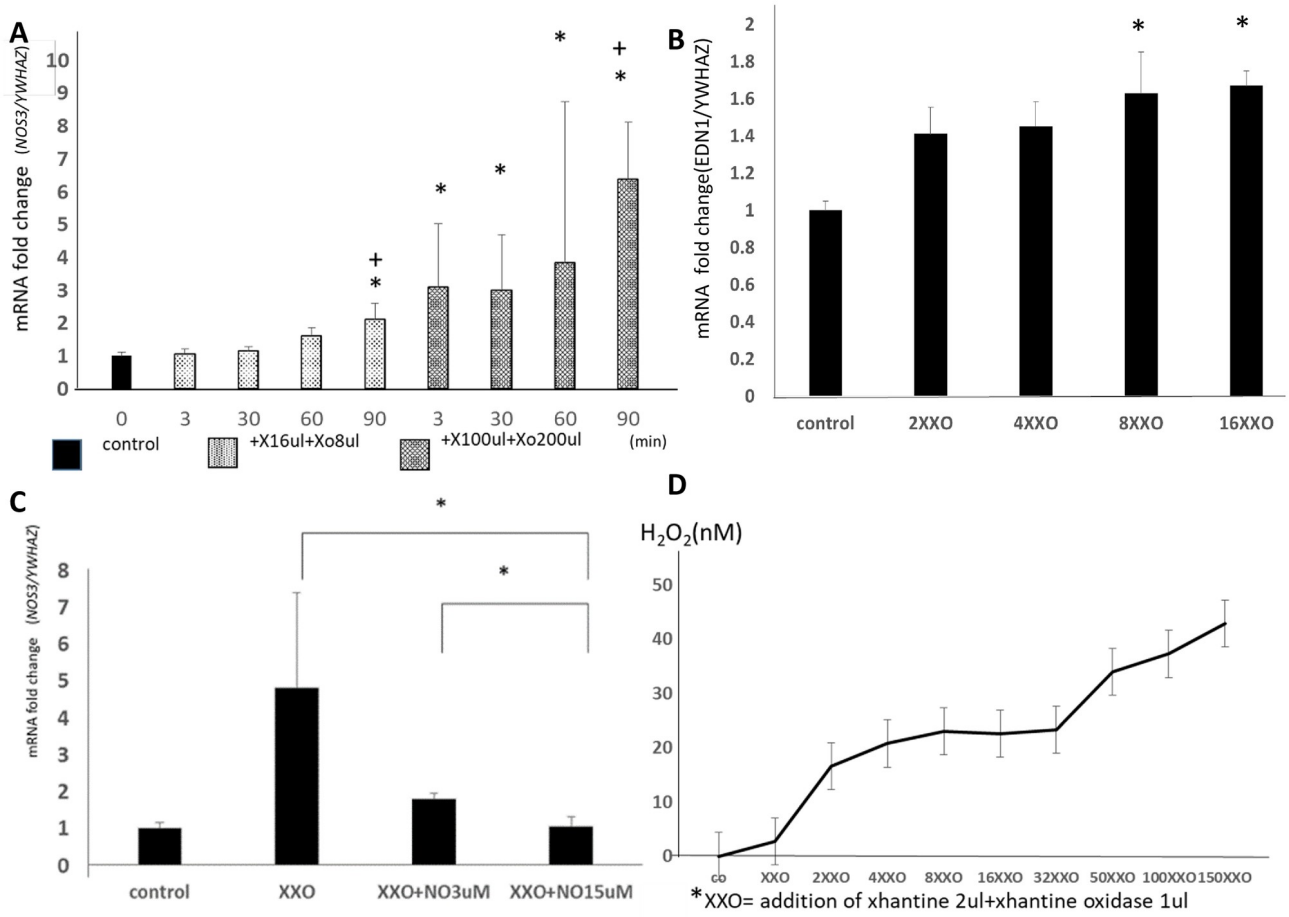
Effect of the administration of xanthine and xanthine oxidase on *NOS3* and *EDN1/YWHAZ* mRNA expression in HUVECs. HUVECs alone were used as a relative control (ratio = 1). HUVECs under low- and high-dose administration of H_2_O_2_ were analyzed at 0, 3, 30, 60 and 90 minutes of co-incubation. *P < 0.05 compared with control; +*P* < 0.05 compared with the 3-minute data in the same dose group. (B) Relative change in *EDN1/YWHAZ* mRNA expression in HUVECs. *P< 0.05 compared with control. (C) Change in relative expression of *NOS3/YWHAZ* mRNA in HUVECs. *P < 0.05 compared with X/XO. (D) Change in H_2_O_2_ concentration in the medium of the cultures administered with increasing doses of X/XO (xanthine 2 μL + xanthine oxidase 1 μL).

We also examined the effect of ROS concentration on *NOS3* and *EDN1* expression in HUVECs. Fig 7A shows *NOS3* mRNA expression at low-level (xanthine 16 mM, xanthine oxidase 500 IU/mL) and high-level (xanthine 160 mM, xanthine oxidase 1,250 U/mL) treatment in H_2_O_2_-producing systems at 3, 30, 60, and 90 minutes of incubation. The longer HUVECs were incubated with both low and high doses of H_2_O_2_, the greater the increase in *NOS3* mRNA levels, although the increase in *NOS3* expression under the high dose of H_2_O_2_ administration was higher than that in the low-dose group.*NOS3* was significantly upregulated by H_2_O_2_ in a dose- and time-dependent manner. However, in the presence of preexisting NO donor in the culture media, the administration of H_2_O_2_ did not induce the upregulation of *NOS3* expression (Fig 8C). Similarly, *EDN1* expression was upregulated by the administration of xanthine and xanthine oxidase in a dose-dependent manner (Fig 8B).

## Discussion

In this study, we show for the first time that stimulated X-CGD neutrophils induced the decreased of *NOS3* and *EDN1* mRNA expression in vitro due to the increased NO and decreased H_2_O_2_ production derived from their neutrophils. Many studies of ROS including O_2_- and H_2_O_2_ revealed that ROS has an influence in the pathophysiology of a variety of vascular diseases [34–36]. It has also been established that ROS has an inductive role in endothelial dysfunction [32]. Among several inducers of ROS, NADPH oxidase is the most important cellular producer of superoxide anion [35]. Leukocyte NADPH oxidase is found in professional phagocytes, neutrophils, and B lymphocytes. However, whether ROS derived from neutrophil NADPH oxidase has a committed role to endothelium cells has not yet been clearly elucidated yet. Violi F. et al. previously suggested that hereditary deficiencies of gp91^phox^, which is a component of NADPH oxidase, associated with enhanced arterial dilatation reflects increasing NO or its bioavailability [11, 36]. In addition, as we also demonstrated in this study, X-CGD neutrophils have also been reported to exhibit increased NO production [9, 10, 37, 38]. Our results, taken together with these previous studies, indicate that it is conceivable that neutrophil NADPH oxidase has a significant effect on endothelial function. Previously, the lack of experimental models, e.g., neutrophils with a *CYBB* knockout, has made it challenging to investigate these interactions in human systems.

To overcome such limitations in this study, we successfully applied two HUVEC culture systems: a cell-free system and a neutrophil-containing system. The transwell system exposed HUVECs to increasing amount of NO metabolites using neutrophils obtained from X-CGD patients without cell-to-cell contact in vitro. In the cell-free system constructed by NO- and ROS-generating reagents, we were able to detect not only changes in gene expression in HUVECs under increased NO or ROS conditions, but were able to replicate the phenomenon that neutrophil-derived NO and ROS could induce endothelial gene expression. We assumed that conditions of NO without ROS in the cell-free system created a similar atmosphere of HUVECs co-cultured with X-CGD neutrophils. In addition, the environment of NO and ROS in the cell-free system also created an environment similar to that with normal neutrophils, namely gp91^phox^.

In the cell-free system, HUVECs under conditions of excess NO without ROS exhibited the downregulation of *NOS3* in a dose-dependent manner. This phenomenon is consistent with previous reports demonstrating that antioxidants such as NADPH inhibitors can reduce eNOS activity in vitro [39, 40]. They have also shown that superoxide anions and H_2_O_2_ are putative inducers of endothelial cell functions in vitro, possibly through upregulation of eNOS activity that leads to increased production of endogenous NO [41]. In agreement with these reports, our findings suggest that excess extracellular NO without ROS such as xanthine oxidase promoted a compensatory reduction in eNOS activity in HUVECs. Interestingly, we found that excess extracellular NO without ROS induced the downregulation of *EDN1* mRNA expression in HUVECs. Previous studies have indicated that various substances, including thrombin, angiotensin II, transforming growth factor-1 and tumor necrosis factor-α, stimulate *EDN1* gene expression in endothelial cells through DNA binding of transcription factors such as activator protein-1 [42, 44]. It has also been reported that *EDN1* mRNA expression levels are downregulated by NO through the NFκB inactivation pathway [43]. Similarly, a negative feedback mechanism involving NO and NFκB that modulates eNOS transcription and protein expression has been detected [21]. Our experiments showed the inactivation of the NFκβ pathway in HUVECs co-cultured with X-CGD neutrophils, consistent with previous reports. In addition, as the expression patterns of *EDN1* and *NOS3* are synchronized in HUVECs, this inactivation of the NFκB pathway may be related to decreased *NOS3* mRNA expression [27].

X-CGD patients have been reported to exhibit significantly higher FMD values [11]. Violi et al. hypothesized that excess NO derived from endothelial cells might induce arterial dilatation through upregulation of *NOS3* in their studies because shear stress has previously been reported to increase *NOS3* expression and eNOS activity in general [11,12]. Previous findings indicating that *NOS3* expression might be upregulated in X-CGD patients would seem to contradict our results. With regard to the phenomenon of higher FMD in X-CGD patients, we speculate that another explanation could be that the effect of H_2_O_2_ derived from ECs dilating other ECs is an endothelium-derived hyperpolarizing factor. Valen et al. reported that H_2_O_2_ induced a NO-dependent vasodilation of coronary flow, and that inhibition of NO is detrimental to left ventricular function after H_2_O_2_-mediated oxidative stress [41]. Our findings also suggest that ROS and antioxidants including NO maintain a constant balance in the vascular system. Therefore, we hypothesized that an excessively high extracellular NO condition derived from neutrophils would be likely to induce H_2_O_2_ from ECs in X-CGD. This mechanism has been speculated for the excessive peroxynitrite derived from extracellular NO that induced tetrahydrobiopterin (BH4) oxidation and eNOS uncoupling [44]. Another speculation involves the effect of endothelin, a vasoconstrictor, which acts in opposition to eNOS. In our study, *EDN1* expression was shown to decrease in parallel with *NOS3* expression, which might explain the mechanism by which X-CGD patients exhibit higher FMD values. Thus, we suggest that NO excess resulting from activated neutrophils with a deficiency of gp91^phox^ is related to arterial dilatation through downregulated expression of endothelial genes such as *NOS3* and *EDN1*.

Conversely, Kirk et al. have reported that X-CGD mice failed to inhibit atherosclerosis [44]. We also found that the X-CGD patients in this study had normal blood pressure, and that non-activated neutrophils from X-CGD patients did not induce significant changes in endothelial *NOS3* and *END1* expression. These results suggest that NO from neutrophils of X-CGD in the absence of stimulation does not have a major function in inhibiting the progression of arterial sclerosis because the concentration is low, similar to that in healthy patients. However, the inhibition of endothelial dysfunction leading to atherosclerosis might occur in X-CGD patients with chronic inflammation.

There were some limitations in this study. The experimental timing and dose conditions for the stimulation of neutrophils. As shown in Fig 1B, slight ROS generation were detected in the supernatant of HUVECs from co-cultured with unstimulated normal and X-CGD neutrophils compared to the supernatant of HUVECs only. We speculated that phenomenon might be caused by laminar flow when neutrophils were added in culture dishes. However, we evaluated those ROS dose did not change significantly between unstimulated normal and X-CGD neutrophils. On the other hand, ROS dose from stimulated neutrophils were detected in Fig 5A as consistent with previous findings.

In addition, ECs in vitro did not necessarily reflect the internal phenomena in vivo [44]. Based on the results in neutrophil-free culture, total eNOS protein expression exhibited a dose-dependent effect according to extracellular NO and H_2_O_2_ concentration. Similar to this phenomenon, non-activated neutrophils of X-CGD might not induce significant changes in endothelial function, including *NOS3* expression. Moreover, we also demonstrated that changes in the phosphorylation of eNOS at Ser1177 is related to the activity of eNOS [17]. Two sites in particular, Ser1177 and Thr495, are related to the activation and inactivation of eNOS, respectively. As shown in Fig 2, phosphorylation at Ser1177 has been shown to lead to the activation of eNOS by sheer stress [45]. It has been also reported that NO directly induced the activation of phosphorylated of eNOS at Ser1177 [46].

In summary, our results provide new findings on the role of stimulated neutrophils in the upregulation of endothelial eNOS and ET-1. Of particular significance is our finding that the gp91^phox^ component of NADPH oxidase in neutrophils is an important molecule related to endothelial gene expression. This novel demonstration that NO and ROS derived from activated neutrophils influence endothelial gene expression will help further efforts to elucidate the interaction between neutrophils and endothelial function. We propose further investigations of the molecular mechanism by which neutrophil NADPH oxidase including gp91^phox^ affects endothelial gene expression, as it could identify potential molecules in the treatment and prevention of endothelial dysfunction.

## Supporting information

S1 Fig Original western blots. Original western blots used to create.
(TIF)

S2 Fig 2-A, 2-C, 3-B, 4-A, 6-B and in the manuscript are provided here as labeled. (PPTX)
