# Supplementary figures and images for: Downregulation of endothelial nitric oxide synthase (eNOS) and endothelin-1 (ET-1) in a co-culture system with human stimulated X-linked CGD neutrophils
Source: PLoS One. 2020 Apr 6;15(4):e0230665. doi: 10.1371/journal.pone.0230665 (PMC7135077; doi:10.1371/journal.pone.0230665)

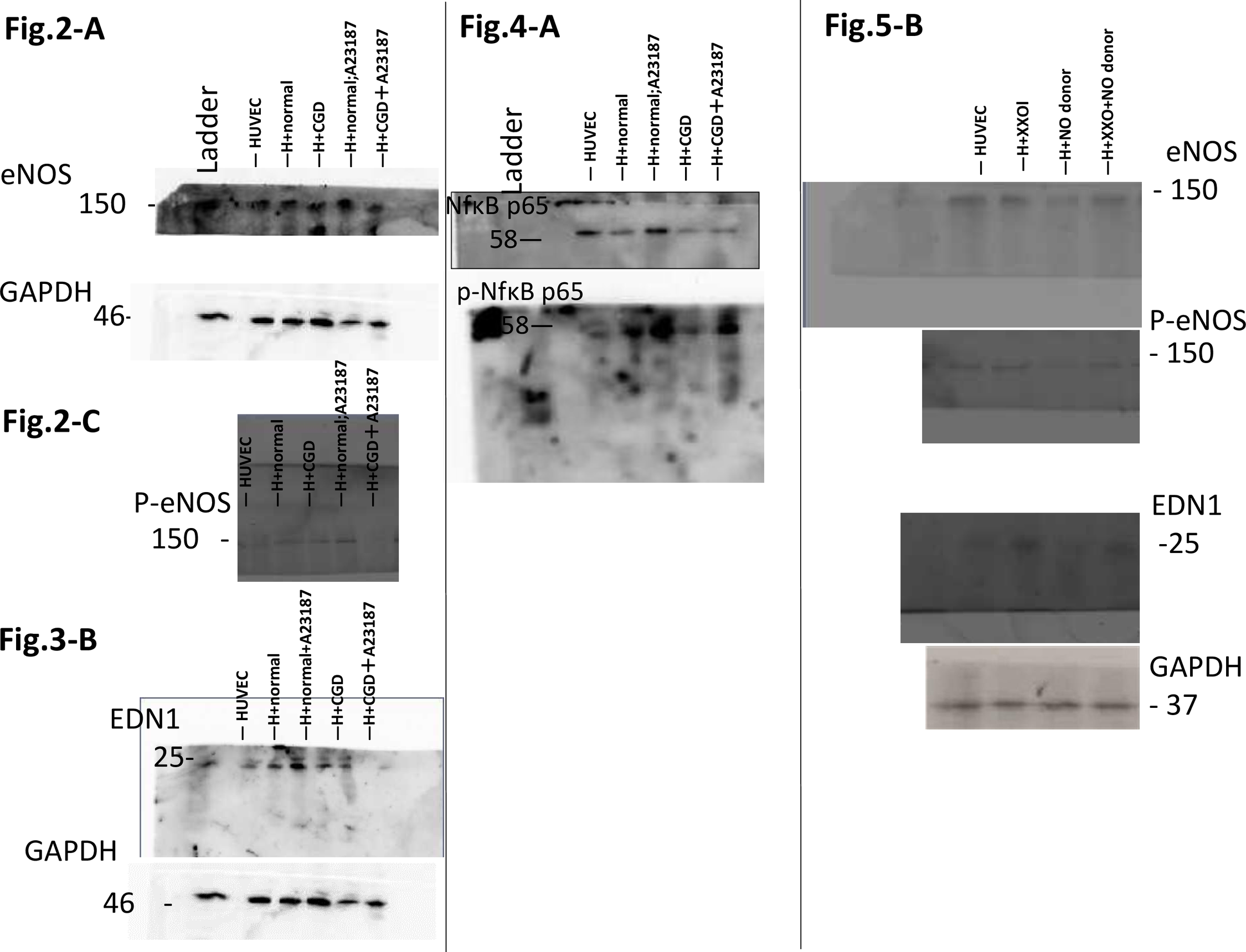

Supplement: S1 Fig — Original western blots used to create. (TIF) [file pone.0230665.s001.tif]
