# Supplementary material for: Downregulation of endothelial nitric oxide synthase (eNOS) and endothelin-1 (ET-1) in a co-culture system with human stimulated X-linked CGD neutrophils
Source: PLoS One. 2020 Apr 6;15(4):e0230665. doi: 10.1371/journal.pone.0230665 (PMC7135077; doi:10.1371/journal.pone.0230665)
Supplement: S2 Fig — (PPTX) [file pone.0230665.s002.pptx]

## Slide 1
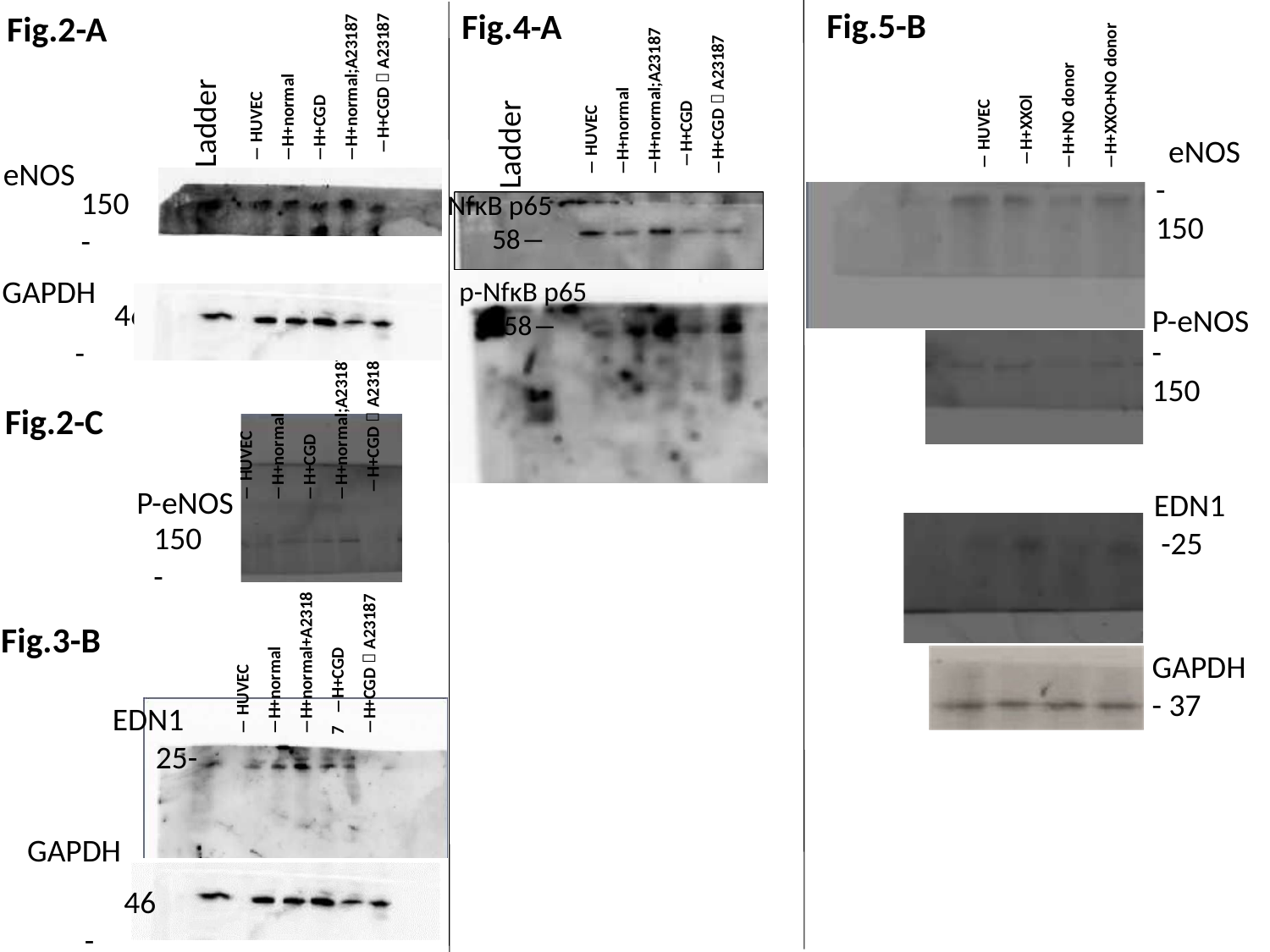

Fig.2-A
Ladder
Ladder
Fig.5-B
Fig.4-A
― HUVEC ―H+normal ―H+CGD ―H+normal;A23187 ―H+CGD＋A23187
― HUVEC
 ―H+XXOl
―H+NO donor ―H+XXO+NO donor
― HUVEC ―H+normal ―H+normal;A23187 ―H+CGD
―H+CGD＋A23187
eNOS
eNOS
- 150
150　-
NfκB p65
 58―
GAPDH
p-NfκB p65
 58―
　46-　-
P-eNOS
- 150
― HUVEC ―H+normal ―H+CGD ―H+normal;A23187 ―H+CGD＋A23187
Fig.2-C
P-eNOS
EDN1
 -25
150　-
― HUVEC ―H+normal ―H+normal+A23187 ―H+CGD
―H+CGD＋A23187
Fig.3-B
GAPDH
- 37
EDN1
 25-
GAPDH
　46 　-
